# Glucose metabolism responds to perceived sugar intake more than actual sugar intake

**DOI:** 10.1038/s41598-020-72501-w

**Published:** 2020-09-24

**Authors:** Chanmo Park, Francesco Pagnini, Ellen Langer

**Affiliations:** 1 Department of Psychology, Harvard University, 33 Kirkland St, Cambridge, MA 02138 USA; 2 Department of Psychology, Università Cattolica del Sacro Cuore, Milan, Italy

**Keywords:** Human behaviour, Metabolic disorders

## Abstract

The authors examine study participants who have Type 2 diabetes to determine whether cognition affects glucose levels in contrast to widely held suppositions. Thirty participants who have type 2 diabetes consume beverages that have identical ingredients but have deceptive nutrition facts labels. Blood glucose levels measured four times before and after beverage consumption show that blood glucose levels increase when participants believe the beverage has high sugar content as portrayed on the labels. Also, individual eating behaviors and nutritional satisfaction are linked to changes in blood glucose levels. The study results support the concept of anticipatory budgeting on glucose metabolism. The findings provide pressing evidence for the psychobiological model of chronic disease, suggesting that psychological intervention programs may be important for diabetes management, beyond current programs in which type 2 diabetes is managed through diet, exercise, and medications only.

## Introduction

There is a growing acknowledgement that both mind and body exert a role in the course of many physical diseases. Several studies suggest that subjective mental states, such as mindsets or expectations, may influence human physiology^1^. For example, our studies have shown that young adults had improved vision when they were exposed to pilot and athlete mindset-primes and when they read Snellen Eye Charts featuring reversed progression^2^, or that expectations to develop influenza-like symptoms during the winter season increase the chances to develop the symptoms^3^.

There is a certain number of mind/body studies that explored the relationship between the psychological domain and food processing and caloric consumption. In a pioneering work, chambermaids primed to view their work as a form of exercise showed weight loss, decreased Body Mass Index (BMI), waist-to-hip ratio, and a drop in blood pressure relative to a control group^4^. In another study, participants consumed milkshakes labeled to indicate higher or lower calories^5^. As a result, peptide ghrelin patterns were then consistent with their perceptions rather than objective nutritional differences. In line with these findings, a recent study demonstrated that believing to follow a low-calorie diet (while in fact having an energy-balanced diet) leads to a body mass reduction^6^. Several papers have identified an anticipatory psychological response following carbohydrate mouth rinse influencing energy budgeting and utilization^7–9^. These findings suggest that the brain in engaged in substantial metabolic and endocrine regulation based on anticipation of challenges and resource availability. This can contribute to an evolutionary advantage, as the expectation prepares the body to better cope with the anticipated event^10^. As blood glucose and insulin signaling is a mean to regulate different organs^11^, the role of anticipatory budgeting mechanisms could be particularly relevant for people with diabetes.

## Psychological influence in diabetic metabolism

The World Health Organization (WHO) reports that diabetes rates have almost quadrupled globally over the past three decades, making diabetes one of the most important international public health challenges causing an estimated 1.6 million deaths in 2015 and costing approximately $825 billion globally per year^12^. Ninety percent of diagnosed diabetes cases are type 2 in which the body fails to generate sufficient insulin or use it properly^6^. Diabetes has short-term and long-term complications, including strokes, neuropathies, kidney disease, and vision problems^13^. While diabetes is generally approached following the dominant biomedical model, there is some evidence that psychological aspects play a role in its physiological processes. For example, stress has been consistently associated with higher blood glucose levels among both nondiabetics and diabetics^14–16^. Moreover, depression^17^ and psychological comorbidity^18^ can affect diabetic physiology.

In our recent work, participants with type 2 diabetes took part in a study that examined whether the perception of time passing affects blood glucose levels^19^. Participants fasted beforehand to ensure similar starting blood glucose levels and consumed no food or drink while playing simple video games, switching games every 15 min, and keeping track of time with clocks provided by the researchers. In all cases, the task period lasted 90 min, but some clocks were rigged to run fast or slow. Unadulterated clocks correctly indicated that 90 min had elapsed; fast clocks reported a total elapsed time of 180 min; slow clocks indicated that only 45 min had passed. All participants spent the same amount of time playing video games, but their time perceptions were altered. Blood glucose samples were drawn from each participant before and after the task period. Although all experienced the same elapsed time, the findings revealed that fasting blood glucose decreases have been associated with the perceived time passage rather than the actual time passage. In addition, participants who thought more time had passed reported being hungrier than those who thought less time had passed. These findings indicate that psychological processes can influence physiological levels, although the role of perceptional and cognitive processes in metabolism is still under-appreciated.

The biomedical model primarily assumes that blood glucose levels rise and fall as time passes after sugar intake. However, as discussed, the subjective perception rather than the objective passage of real time has been shown to determine blood glucose level changes in people with type 2 diabetes^19^. In the current study, we targeted the amount of sugar consumption, which is the most widely accepted factor in explaining blood glucose fluctuations, to investigate whether psychology mediates the effect on blood glucose levels in people with type 2 diabetes. We used a food label method similar to that used by Crum and colleagues^5^. Our study participants drank identical beverages containing the same amount of sugar level, but they were labeled as having either higher sugar or lower sugar. We hypothesized that perceived rather than actual sugar consumption would influence blood glucose levels. In addition, we aligned the study with empirical evidence regarding psychological factors that influence blood glucose levels such as perceived hunger^19^, stress and mood^18^, restrained eating behaviors^20^, and nutritional satisfaction^21^. We also searched for potential idiosyncratic factors that may influence blood glucose levels as well as potential mediators or moderators affecting the relation between perceived sugar consumption and blood glucose levels.

## Methods

### Participants

We used flyers and local advertisements to recruit volunteers who have insulin-independent type 2 diabetes mellitus and were being treated with diet and metformin, a biguanide antidiabetic medication. We advertised our “Beverage Tasting Study for Diabetes” study as a clinical investigation at Harvard University in exchange for travel expenses and $70 for two 1.5-h sessions. The advertisement stated: “We are interested in the effects of specially designed beverages on the body's reaction and cognitive functioning among people with type 2 diabetes”. At least three days before participants came to the laboratory, they received a package of forms and instructions, including a brief survey about their medical conditions, a daily glucose diary, a glucose fluctuation chart, and fasting instructions. To ensure that they would be familiar with their own BGL fluctuations, participants were instructed to record their blood glucose levels before and after every meal and to complete a daily blood glucose change chart for three days before the experiment. To minimize potential BGL variability, we asked participants to fast for at least 8 h before the study, which began at 9:00 AM. During the prescreening medical period, we excluded volunteers whose average fasting blood glucose levels were over 200 mg/dL (or 11 mmol/L) to minimize the potential risk of postprandial hyperglycemia related to the beverage tasting task.

The present study was approved by the Institutional Review Board (IRB) for protection of human subjects in research at Harvard University. The protocol was reviewed and registered by the Institutional Biosafety Committee (IBC) at Harvard University, *the Committee on Microbiological Safety* (COMS). All the research staff involved in handling human blood samples were trained before the project began, in compliance with COMS policies. All methods were performed in accordance with the approved guidelines and regulations, and all participants provided written informed consent. The study protocol was registered in the ISRCTN registry (ISRCTN81937091, dated 03/06/2019).

Initially, we recruited thirty-four participants, but three failed to attend the second session, and one failed to follow the fasting procedure. After excluding their data, we analyzed data for the remaining thirty participants (≥ 12-month duration since diagnosis; 47% women; mean age = 52.13 years, SD = 2.26 years; 50% White, 36.7% African American, 6.7% Asian, and 6.7% other; mean fasting hours: 11 h 38 min; SD = 1 h 28 min).

### Design and procedure

**Figure 1:**
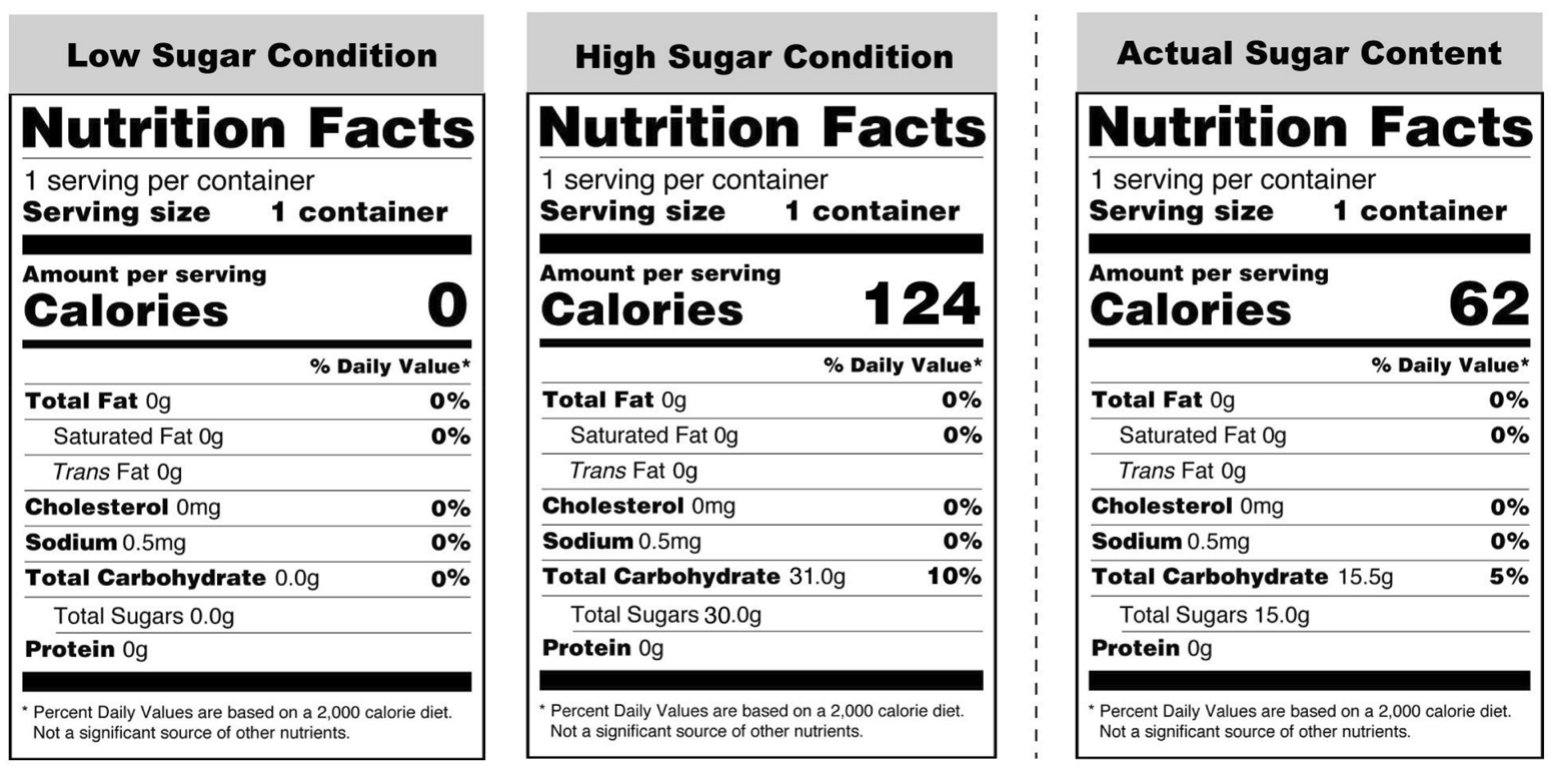
Two labels used in the study (left) and actual nutrition contained in the beverage (right).

In a within-subject design, participants were instructed to come to the laboratory twice, with a three-day interval between visits. When participants first arrived, we explained that we were gathering evaluations of the taste and perceived nutritional value of a high-sugar beverage and a low-sugar beverage designed for people with type 2 diabetes (see Fig. 1). At each session, participants consumed one of the two beverages, which were actually identical but had labels indicating different sugar levels (Label 1: 0 g sugar, Label 2: 124 g sugar, Actual: 62 g sugar). We counterbalanced the order of presentation based on a block randomization procedure, creating two equally-sized group samples. Instructions and surveys were presented using the Qualtrics survey software package (Qualtrics, Provo, UT).

After participants signed informed consent forms, they filled out a series of baseline questionnaire items. After a researcher measured and recorded starting blood glucose levels, participants consumed and evaluated their assigned beverages, some labeled as having high sugar (124 g), others labeled as having low sugar (0 g). We controlled for consumption speed by instructing participants to completely consume the beverage in 3 min. We then measured blood glucose levels three times, with an interval of 20 min from the baseline measurement. After the final blood glucose measurement of the second session, participants were debriefed and compensated.

### Measures

#### Blood glucose

Researchers were trained to handle biological wastes (e.g., blood) and sharps (needles) by the Committee on Microbiological Safety (COMS) at Harvard University and strictly followed its regulations. Blood glucose levels were measured by the Bayer Contour Next EZ^22^. We analysed 240 blood glucose measurements. Participants’ mean fasting baseline blood glucose level was 143.7 mg/dL (SD = 32.6).

#### Beverage evaluation

Participants received a form for evaluating the beverages according to taste, nutritional value, and overall enjoyment. They also indicated whether they would drink the beverage regularly, and if so, why. As a manipulation check, participants rated their perceived sugar level of the beverages they drank from 1 (*very low*) to 5 (*very high*). The participants also rated their satisfaction with the nutrition facts on the beverage labels from 1 (*very dissatisfied*) to 5 (*very satisfied*).

#### Perceived stress and eating behaviors

The pre-intervention surveys included the Perceived Stress Scale (PSS, 10-item version; α = 0.78)^23^ to assess baseline levels of individual stress. At the end of the first session after the final blood glucose measurement, we assessed individual variations of eating behaviors through the Dutch Eating Behavior Questionnaire (DEBQ)^24^, which includes three subscales to assess restrained eating (10 items; α = 0.95), emotional eating (13 items; α = 0.94), and external eating behaviors (10 items; α = 0.80).

#### Momentary stress, affectivity, and hunger

The PSS scale primarily assesses stable perceptions of stress over prolonged periods, so we also included a Single Stress-Measuring (SSM) question (item endpoints: 1, *not stressed at all* to 10, *extremely stressed*), and the Positive Affect and Negative Affect Scale (PANAS)^25^. Cronbach's alphas of PANAS were 0.88 for the 10 positive affect items and 0.87 for the 10 negative affect items. Finally, we used the Satiety Labeled Intensity Magnitude (SLIM)^26^, a single vertical line scale with an average reliability coefficient of 0.90, to examine whether the beverage type and the study order influenced subjective hunger during the tasks. We administered the SSM, PANAS, and SLIM at the pre-intervention session, again at the mid-intervention session (after the second blood glucose measurement), and finally at the post-intervention session.

## Results

### Perceived sugar intake

**Figure 2:**
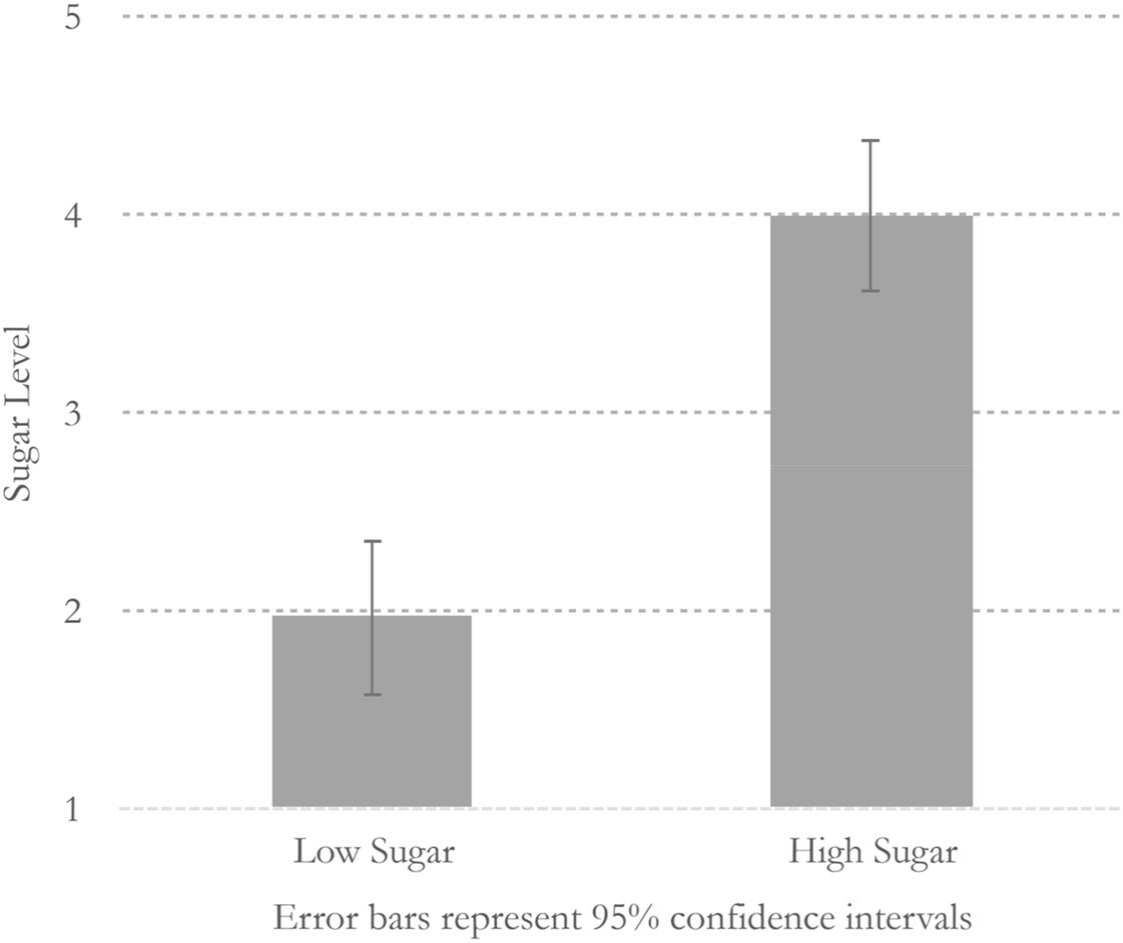
The effect of the label manipulation on perceived sugar intake.

To assess the effect of the label manipulation on perceived sugar intake, we fit a linear mixed model, with label type (high/low sugar) and study order (high sugar label in the first session vs. low sugar label in the first session) interaction terms as fixed effects. Random intercepts were set for individual subjects in each label type, to control by-subject variation and by-manipulation variation in the model. Beverage type had a significant main effect (*β* = − 2.06, SE = 0.38, *p* < 0.000), but order or the interaction effect between the beverage and the order had a nonsignificant main effect (*p*s > 0.9). Figure 2 shows effects of the label manipulation on perceived sugar intake. Simple effect tests suggested a significant difference in perceived sugar intake as a function of the beverage label; low sugar condition (mean = 1.97, SD = 1.10) vs. high sugar condition (mean = 4.00, SD = 1.02), *t*(58) =  − 7.44, *p* < 0.000, *d* = 1.92.

#### Momentary stress, affectivity, and hunger

To test whether beverage type, study order, and blood glucose measurements systematically induced momentary stress (SSM), positive or negative affectivities (PANAS), and subjective feelings of hunger (SLIM), we performed a mixed model analysis of variance (ANOVA) by including order as a between-subject factor and beverage type and time as within-subject factors in the model. Results indicated no significant main or interaction effects for all three measures (*ps* > 0.17).

#### Blood glucose level

**Figure 3:**
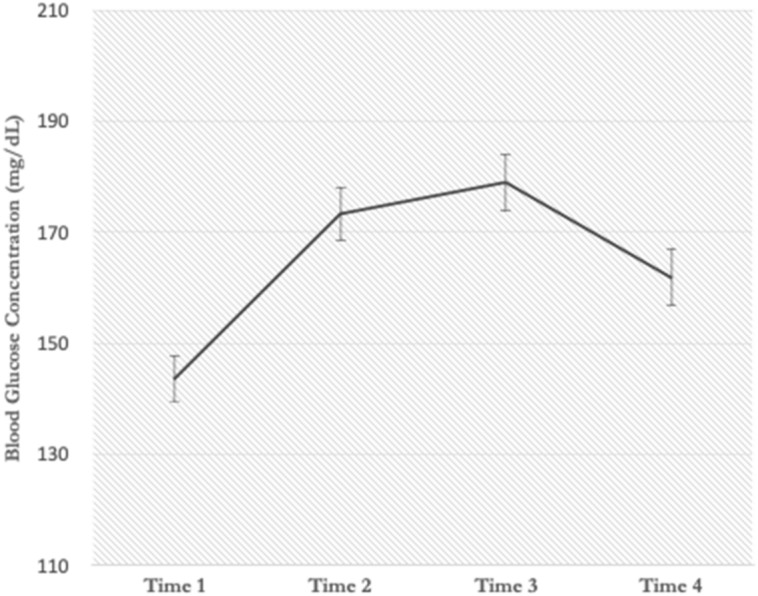
Average blood glucose levels over time.

Figure 3 graphically represents average blood glucose levels for participants across four time points. We fit linear mixed models by incorporating into the model time (baseline, 20 min, 40 min, and 60 min), beverage type, and order (with all interaction terms) as fixed effects and random intercepts for subjects and beverage type. Order effects were nonsignificant (*p*s > 0.25). In fact, a model with beverage order removed was an improved fit over the previous model, χ^2^ (1) = 2094.72–2086.82 = 7.9, *p* < 0.01. Main effects for time were all significant, as expected (*β*_*t2*_ = 16.03; *β*_*t3*_ = 20.97; *β*_*t4*_ = − 23.00, SE_*all*_ = 3.75, *ps* < 0.000). Furthermore, as predicted, beverage type significantly interacted with time 2 and time 4 (*β* = − 9.13, SE = 4.00, *p* = 0.025 and *β* = 9.7, SE = 4.00, *p* = 0.017, respectively). Beverage type interacted with time 3 in a marginally significant fashion (*β* = − 7.60, SE = 4.00, *p* = 0.061).

**Figure 4:**
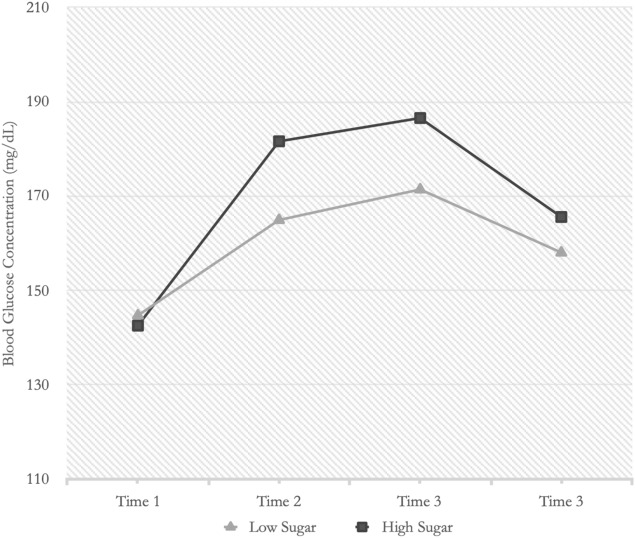
Average blood glucose levels over time as a function of beverage type.

As Fig. 4 shows, label type exhibited a curvilinear effect on blood glucose levels over time. To test for nonlinear blood glucose changes, we compared a model fit with a quadratic term to the original model with the linear time parameters. The quadratic model fit showed an improvement over the linear model using the Akaike Information Criterion (AIC) (Δ AIC = 2,118.11 vs. 2,116.74) but not using a Likelihood Ratio test (*p* > 0.05). However, for subsequent analyses, we used the quadratic model, due to its AIC fit and because it matched our hypothesized pattern of blood glucose responses.

### Perceived stress and eating behaviors

To examine whether perceived stress over the past month influenced how beverage type affected blood glucose levels, we entered PSS as a covariate in the final model. Interaction effects involving PSS had nonsignificant main effects (*ps* > 0.4), but beverage type no longer had a main effect at the significance level of α = 0.05 (*p* > 0.3), when PSS was controlled. In other words, perceived stress was not directly linked to any factors in the model to predict blood glucose levels, but it accounted for substantial overall effects of beverage type on blood glucose levels.

To check whether individual eating behaviors were related to the label manipulation effect on blood glucose levels, we added the restrained, emotional, and external eating subscale score for DEBQ as covariates in the final model. For restrained and emotional eating behaviors, no main effects or interactions occurred with any predictors (*ps* > 0.1). However, beverage type no longer had a significant main effect for emotional eating (*p* > 0.84), similar to the pattern observed when we controlled for PSS. External eating behaviors, however, significantly interacted with beverage type (*β* = − 14.97, SE = 5.83, *p* < 0.05), with time 2 (*β* = − 15.03, SE = 5.31, *p* < 0.01), and with time 3 (*β* = − 10.95, SE = 5.31, *p* < 0.05). When we controlled for external eating, beverage type had a nonsignificant main effect (*p* > 0.9), indicating potential mediating effects on the relation between beverage type and blood glucose levels.

### Nutritional satisfaction and external eating behavior

To assess whether perceived satisfaction with nutrition facts (NS) influenced the effect of label manipulation on blood glucose levels, we entered NS as a covariate in the final model. NS significantly interacted with beverage type (*β* = − 11.40, SE = 4.54, *p* < 0.05) and time 2 (*β* = − 7.66, SE = 3.04, *p* < 0.05).

To clarify how NS affected the relationship between the label manipulation and blood glucose levels, we performed a serial mediation analysis testing how beverage type indirectly affected blood glucose levels through the potential mediators based on the bootstrapping procedure^24^. Furthermore, to identify how external eating behaviors were linked to blood glucose levels directly or indirectly through our predictors, we performed a simple mediation analysis for the external eating variable using the approach described above.

**Figure 5:**
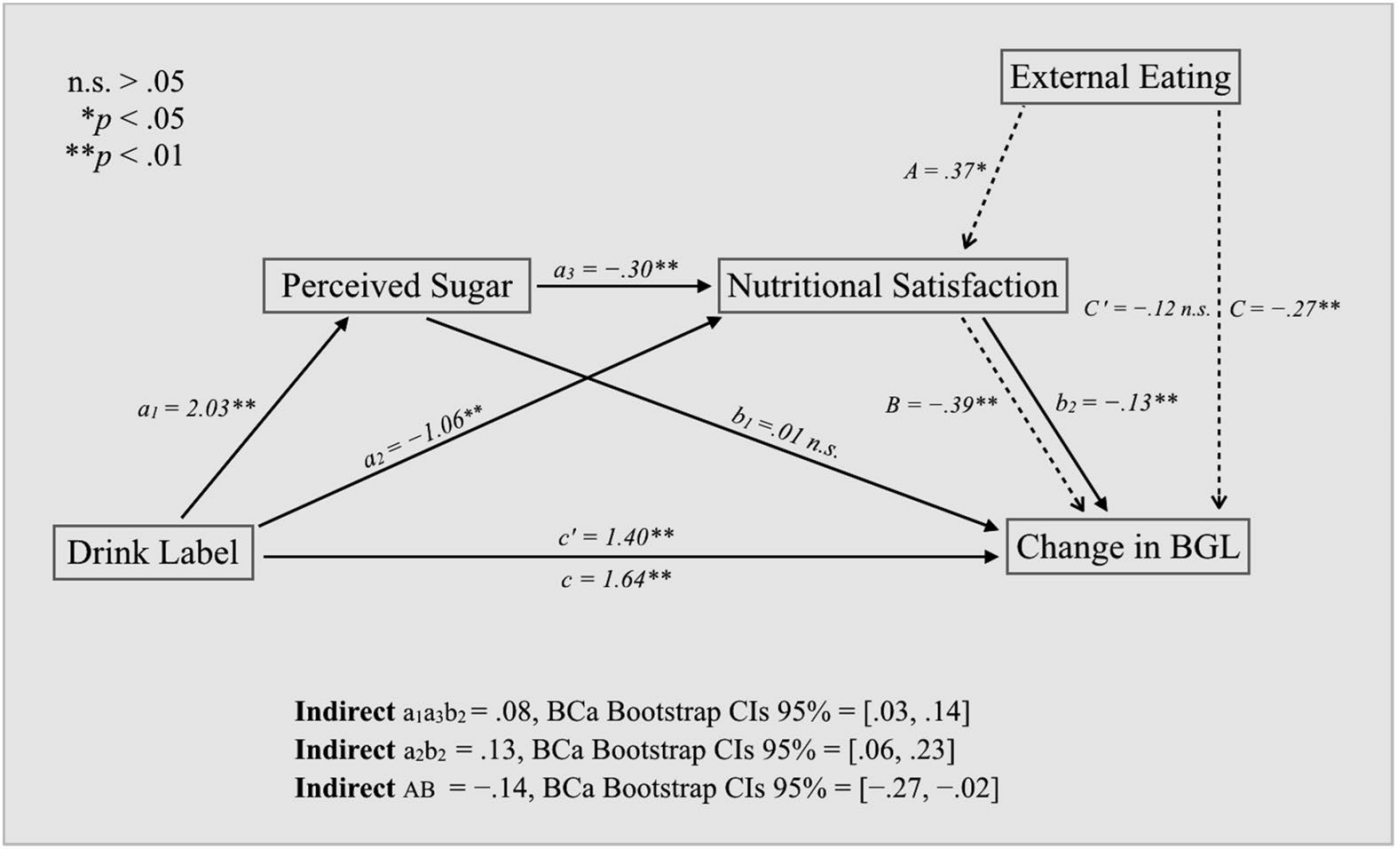
Path diagram illustrating how perceived sugar and nutritional satisfaction serially mediate the relationship between beverage type and changes in blood glucose levels and how nutritional satisfaction mediates the relationship between external eating behaviors and change in blood glucose levels. All presented path coefficients were unstandardized. The unit for the change rate of blood glucose level used in the mediation analysis was mg/dL per minute.

Figure 5 shows a path diagram illustrating direct and indirect effects for two mediation models and causal paths linking beverage type with blood glucose changes, along with the 95% Bias-Corrected and Accelerated Bootstrapped Confidence Intervals (BCa Bootstrap CIs) for path estimates. The two mediation analyses revealed that two sequential mediators—perceived sugar intake and nutritional satisfaction—partially mediated the effect of beverage type on blood glucose levels (Ba1a3b2 = 0.08, SE = 0.03, 95% bootstrapping CI 0.03–0.14), and that external eating behavior is indirectly related to blood glucose levels through its relationship with nutritional satisfaction (BAB = − 0.14, SE = 0.06, 95% bootstrapping CI − 0.27 to − 0.02).

## Discussion

In this study, we tested whether psychological factors influence the effects of sugar consumption on blood glucose levels in people with type 2 diabetes. We hypothesized that people with type 2 diabetes would show significant blood glucose responses to their perceptions of sugar consumption. Although beverages used in all study sessions contained the same sugar content, the results showed blood glucose profiles aligned more with what study participants had actually believed from the label of sugar contents (see Fig. 2). That is, blood glucose values changed according to the participants’ expectations (either low or high sugar content), and not to the amount of sugar they actually consumed.

These findings challenge the mainstream assumption that natural biological and physiological metabolic homeostasis processes require sufficient insulin to allow glucose to return to normal ranges. This is in line with other works that found no evidence that insulin action determines the steady-state level of glucose^27^. In contrast to conventional biomedical models and their assumptions of independent actions of the physiological processes, we show that subjective perceptions of sugar intake, even when incorrect, produce measurable biochemical changes in diabetic metabolism. To understand the results, we must consider psychological dimensions working on the relationship between physical stimuli and bodily responses.

It is, however, important to note that our study does not indicate whether psychological effects have long-term efficacy. Our findings instead suggest that psychological processes may be, at least temporarily, able to influence biochemical processes in diabetic metabolism. The excess of blood glucose occurring in hyperglycemia, together with the atypical performance of pancreas, however, fits well into the anticipatory budgeting mechanism. Emotions and mental states are linked to the autonomic nervous system, specifically the parasympathetic/sympathetic responses to blood glucose. Perceptions of sugar level would produce altered glucose dynamics as they provide a different signal to pancreas. A hyperglycemic response can be then understood as an excessive anticipatory energy budgeting following glucose perceptions. Additional research into the physiological or biochemical mechanisms underlying these psychological effects and their long-term effects is necessary to further refine the biopsychosocial model of diabetic metabolism.

In addition to these results, we find some intriguing individual factors. When we controlled for stable idiosyncratic factors such as emotional eating habits and the level of perceived stress, perceived sugar intake had significantly less effect on blood glucose levels. As Fig. 5 shows, the indirect pathway a_2_b_2_ (drink label → nutritional satisfaction → blood glucose) bypasses perceived sugar consumption to have an even stronger effect on blood glucose changes than the indirect pathway a_1_a_3_a_2_, which passed through perceived sugar intake (drink label → perceived sugar → nutritional satisfaction → blood glucose). Perceived sugar intake, however, was not directly related to the changes in blood glucose levels. Accordingly, affective feelings associated with the nutrition facts seem to have a pivotal role in determining blood glucose changes in our model. It is possible that people with diabetes who decide whether to eat based on external stimuli (e.g., when it’s time to eat, or when other people eat), rather than based on internal stimuli (e.g., feelings of hunger), react more strongly to the labels on the beverages.

Our mediation analysis also revealed that the individual factor of external cued eating is linked to changes in blood glucose levels (Fig. 5). In contrast to internally directed eaters, externally cued eaters are heavily influenced by factors such as visual and olfactory cues^24^. Consequently, especially those who were diagnosed with diabetes at least 12 months ago may be more aware of nutritional facts and, thus, form strong food perception from reading nutrition labels. In fact, in contrast with the suggestions from the developers of the DEBQ scale, our participants had an overall mean score of external cued eating (mean = 3.25; SD = 0.67), which is much higher than the estimated mean score of the people without diabetes (mean = 2.65; SD = 0.54) or even the mean score of the obese patients^24^ (mean = 2.71; SD = 0.60). These findings provide an interesting new direction for future research in diabetes management. If external cued eating is an individual factor that mediates the relationship between perceived sugar intake and blood glucose levels, it would be worthwhile to examine a possibility that the high glycemic variability in type 2 diabetes is directly linked to the idiosyncratic strength in their association between feelings about nutrition facts and expectations about blood glucose response.

Our study has limitations that must be addressed. First, participants were required to consume a beverage containing sugar. Therefore, we excluded volunteers whose average fasting blood glucose level was over 200 mg/dL (or 11 mmol/L) during the prescreening medical period to minimize potential risks of postprandial hyperglycemia. Consequently, we must be cautious in generalizing our findings to the full scope of the type 2 diabetic population. Currently, no data are available to accurately compare our sample to the overall population. Based on the data gathered from the previous study^4^ and the current study, however, more than 96% of type 2 diabetics reported fasting blood glucose levels of less than 200 mg/dL during the medical screening processes, suggesting that our sample likely reflects the general population of type 2 diabetics.

In addition, our study took place in a psychology laboratory on a school campus rather than in a hospital or medical school. Some participants indicated that the study location made them doubt whether our procedure was legitimately aligned with our advertised purpose. We asked all study participants to indicate what other purposes might be behind our study at the end of the final session, however, and they were unable to identify our purpose or manipulations. However, as described earlier (Fig. 2), participants in the sugar-free condition still believed that the beverage they consumed contained some sugar. We suspect their suspicions might have had a negative impact on our model’s overall power to detect the effect of perceptions. If so, the results could actually be even stronger. Nonetheless, we used altered nutrition facts mainly to induce two contrasting beliefs about the identical beverage, and the manipulations successfully produced the contrasting beliefs, despite the non-zero mean rating of perceived sugar levels on the sugar-free beverage. Future researchers who use a similar food-labeling method, however, might better detect the effects of perceptions by carefully choosing a study location more consonant with the participants’ expectations. Finally, future studies can be benefit by including a non-treatment group, or use a design that manipulates actual sugar contents to compare psychological and physiological effects.

## Conclusion

Our study indicates that blood glucose level in people with type 2 diabetes is influenced by the perception of sugar consumption. Blood glucose levels increased in accordance with how much sugar participants believed they consumed rather than how much they actually consumed. These findings clearly show the inadequacy of the classical pathways to explain the metabolic and physiological reactions to food intake in diabetics suggested by the biomedical framework. Similarly, recent studies of chronic diseases, as well as on aging^28,29^, are consistently revealing the undeniable influence that psychological processes exert on various chronic physiological and biochemical conditions including diabetes^19^, cardiovascular disease^30^, and chronic obstructive pulmonary disease^31^. In the face of rapidly surging epidemiological patterns of noninfectious fatal chronic diseases, we hope that our efforts to return the mind *back* to the equation of the dominant biomedical formulae will help stimulate more research endeavors in the biopsychosocial field. The goal is to find more effective treatments for millions who have resigned to feeling helpless in the battle against uncontrollable biological processes causing illness and disease, perhaps by recognizing that the mind has meaningful control in regulating health.

## Data Availability

The data that support the findings of this study have been deposited in Harvard Dataverse with the identifier (https://doi.org/10.7910/DVN/2WC8LC).
